# A cross-sectional survey of internet use among university students

**DOI:** 10.1007/s00406-020-01211-1

**Published:** 2020-11-16

**Authors:** Kristina Adorjan, Simon Langgartner, Maximilian Maywald, Susanne Karch, Oliver Pogarell

**Affiliations:** 1 Department of Psychiatry and Psychotherapy, Institute of Psychiatric Phenomics and Genomics (IPPG), University Hospital, LMU Munich, Nussbaumstr. 7, 80336 Munich, Germany; 2 Institute of Psychiatric Phenomics and Genomics (IPPG), University Hospital, LMU Munich, Munich, Germany

**Keywords:** Internet, Addiction disorders, Behavior, Students

## Abstract

The last 2 decades have seen an increase in the number of reports of excessive internet use. Therefore, this study aimed to examine internet use among university students to gain more insight into the novel phenomenon of addictive internet use (AIU). Data were collected by the means of an online questionnaire sent to 4391 students. Approximately 10% of the 4391 students could be included in the statistical analysis. Of those 483 students, almost all (99.2%) used the internet, and a quarter (24.8%) showed AIU. The students used the internet mostly for information searches, random browsing, social networking, and online shopping; however, AIU was seen most often in the areas of social networking, random browsing, information searches, gaming, and pornography. One in four of the respondents showed addictive behavior in at least one area of internet use. Students with AIU in the area of random browsing were significantly less far advanced in their studies than those without AIU, and well-being was significantly poorer across AIU groups than in those who did not show AIU. The study confirms the importance of AIU, as reflected in the high prevalence of AIU among the students and the significantly lower level of well-being in those with AIU. Undifferentiated consideration of AIU does not do justice to its various facets, and future research should consider all areas of internet use, with the aim to increase understanding of the underlying mechanisms of AIU and develop more differentiated treatment approaches.

## Introduction

In 2019, Europe had 727,559,682 internet users, corresponding to 87.7% of the total European population [37]. The majority of users primarily use the internet for social interactions, work, and leisure [4]. However, as the importance of the internet has grown over the past 2 decades, so have the reports of its negative consequences [6]. In Germany, the number of people seeking advice on addictive internet use (AIU) has also increased [99]. In recent years, many different groups studied the phenomenon of AIU, which led to discussions about including it in the diagnostic classification systems. Internet gaming disorder is now included for the first time in the new edition of the Diagnostic and Statistical Manual of Mental Disorders, DSM-5 (as a condition for further study; [1].

On a psychological level, some people use the internet as a reward or coping strategy to overcome emotional crises or dissatisfaction, and such use is not necessarily primarily dysfunctional [20, 22, 35]. For example, almost one third of people name this as the reason why they play online games [35]. A moderate amount of online gaming can even have a positive effect on visual short-term memory [102]. Although these positive aspects apply to most internet users, negative effects are found in some people. For example, six out of ten pupils endanger their school or work performance through their internet use [52] and use the internet as a coping strategy [11] in the sense of an emotionally focused coping style [60]. Some people develop AIU, which can have a negative impact on interpersonal, social, and academic functioning and is also associated with several psychiatric problems, such as depression, anxiety disorders, and sleep disorders [59, 97]. Neuroscience research showed that AIU can also have a negative impact on identity formation [43] and cognitive performance [78] in adolescence and can lead to structural changes in the central nervous system [67, 108, 109].

Similar to substance use disorder, AIU is associated with symptoms such as tolerance development, withdrawal, and craving [26], and some authors view AIU as a behavioral addiction [32, 53]. AIU is also associated with impulse control disorders [47, 91, 105] and shows similarities to pathological gambling [3, 73, 89]. AIU is not associated with age, level of education, social status, or financial means [106]. However, the prevalence of AIU varies considerably depending on the sociocultural background, cohort, and diagnostic tool used. For example, a study in Greece found that 1.5% of adolescents were affected [50]; a study in China, 5% [24]; a study in South Korea, 10.7% [79]; and a study in Germany, 11.3% [74]. Age is also associated with the prevalence of AIU: a study in South Korea found a prevalence of 12.5% in adolescents and 5.8% in adults [58]. A representative study in the general German population (aged 14–90 years old) found a relatively low prevalence of AIU of 0.2%; this finding was likely partly due to the broad age spectrum and partly to the study’s high diagnostic threshold [20]. Studies in Asia found high prevalence rates among students of 12.8% [110], 15.2% [14], and 15.3% [68]. Adolescents [65] and school students [101] are at risk of AIU, and young adults who play online computer games are predominantly single and still live at home [30]. A systematic review of studies on internet and game addiction found that 1.5% to 3.5% of adolescents and young adults are addicted or at danger of becoming addicted [82].

Potential risk factors for AIU include personality traits, reason for internet use, structural requirements of the respective area of use [55], and environmental factors [87]. Personality traits such as extraversion, emotional stability, agreeableness, negative valence, and attractiveness also seem to play a role in the development of AIU [13]. Adolescents with AIU are significantly more impulsive [16, 76, 96] and aggressive [41, 62, 72] than adolescents without AIU. AIU is also associated with social self-efficacy, loneliness, and low intrafamilial interaction [38, 87], poor social skills [23], high interpersonal sensitivity [42] and social withdrawal [87]. Longitudinal studies found that aggression, anhedonia, and emotional problems were important predictors for AIU [31, 94, 95]. Reasons for using the internet that can be risk factors for AIU include dealing with negative emotions [30, 36], dissociation [5], entertainment [5], virtual friendships or relationships [5, 9], playfulness and loyalty [70], curiosity and commitment [33], reward [33], and immersion [9].

Similarities can be found between substance-use disorders and AIU not only at the behavioral level but also at the brain-structural level [61]. In voxel-based morphometry studies, various working groups found reduced grey matter density in people with AIU in the dorsolateral prefrontal cortex, supplementary motor cortex, orbitofrontal cortex, cerebellum, and left rostral anterior cingulate cortex and reduced white matter density in the left anterior cingulate cortex, left posterior cingulate cortex, left islet, and left lingual gyrus [98, 109, 111]. Several of these brain areas are associated with the development of addictive or compulsive behavior. Atrophy of the gray matter of the prefrontal cortex is associated with loss of control: the orbitofrontal cortex regulates impulse control, and the dorsolateral prefrontal cortex and rostral anterior cingulate cortex are associated with cognitive control [51]. An increase in cortical thickness was seen in people with AIU in the left pre-central cortex, precuneus, mid-frontal cortex, and middle and lower temporal cortex [108]. The precuneus is involved in visual processing, attention, and memory recall and is, therefore, known as an area that plays a role in stimulus-induced desire [12]. The lower and middle temporal cortex are also involved in stimulus-induced cravings, so the increase in thickness in these brain areas can be linked to this phenomenon in AIU [46]. Diffusion tensor imaging studies showed less fractional anisotropy in individuals with AIU than in a healthy control group in different areas of the brain, including the white matter in the orbitofrontal cortex and corpus callosum, indicating less anatomical connectivity in the AIU group [67]. In functional magnetic resonance imaging studies, people with AIU showed increased brain activity in the left orbitofrontal cortex and bilateral caudate nucleus in Go/No-go tasks. This increased activity correlated positively with the measured impulsivity [44]. These changes could explain the loss of control in people with AIU [77]. In addition, stimulus induction studies in people with AIU showed greater activity in the right orbitofrontal cortex, right nucleus accumbens, medial frontal cortex, right dorsolateral prefrontal cortex, and nucleus caudatus, and this activity correlated with the subjectively experienced urge to play an online computer game [45]. This activation pattern is similar to that in people with substance addiction who express a strong desire to use the substance [21]. Positron emission tomography studies showed dopamine imbalances in people with AIU while they played a computer game, i.e. increased dopamine release and binding [77]. This release and binding behavior was similar to the injection of stimulants such as amphetamine or methylphenidate [49].

People with AIU show dysfunctional internet use in different areas [107]. Some shop excessively online, some gamble excessively, and some spend hours researching irrelevant information [103]. Online computer games [56], pornography [27], gambling [29], and social networks [54, 66] are common areas of AIU. Random browsing is also a problem for some users [88], and computer games have a high addiction potential [34, 55, 64]. The profile of people with AIU differs, depending on the area of use. For example, users of online computer games are primarily male [84] and users of social networks are primarily female [85]. Because large differences are found between the characteristics of people and the prevalence of AIU for different areas of internet use, the various areas need to be studied separately [85]. Almost two decades of research on AIU indicates that students are a risk group. Therefore, the aim of this study was to evaluate internet use in a group of university students to determine which areas of the internet are relevant for AIU, whether certain features are characteristic of AIU, and whether AIU has any negative consequences.

## Materials and methods

The study was approved by the ethics committee of the medical faculty of the LMU. The data were acquired with an online questionnaire that was distributed by e-mail via the university mailing list of the LMU information service. The questionnaire was sent to students in the winter semester 2012/2013 who had previously agreed to participate in surveys, i.e. to 4391 of the 48,944 students. Participation in the survey was voluntary and anonymous. Inclusion criteria were enrolment at the LMU, and agreement to receive the study materials from the information service. The exclusion criterion was failure to complete the questionnaire beyond the section on demographic variables.

### Assessment instrument

A large problem in research on AIU is the use of many different assessment instruments, e.g., the Compulsive Internet Use Scale [71], Young's Internet Addiction Test [107], Assessment of Internet and Computer Game Addiction Scale [104]. Although the various instruments differ, they contain several common items related to diagnosing an addiction syndrome according to ICD-10. The heterogeneity of the instruments makes it difficult to compare the results of studies, so we decided to classify addictive behavior on the basis of the ICD-10 criteria. To assess both internet use and AIU, we embedded the ICD-10 criteria in a self-designed German-language questionnaire containing 137 questions, which were subdivided into the following sections: sociodemographic characteristics, general internet use, information searches, random browsing, gaming, social networking, online shopping, online pornography, online gambling, substance use, and well-being. The questionnaire was digitalized with the software Lime Survey, version 1.92 + Build 120725, and accessed via an online link. This software ensures anonymity by storing the access key separately from the dataset and allowed us to use a variety of question types with different answer modalities: simple answers (*n* = 113 questions); free text answers (*n* = 19), multiple answers (*n* = 3), and matrix questions (*n* = 2).

### Classification of addictive behavior

To assess internet use in the various areas, we adapted the above-named 6 ICD-10 questions used for diagnosing a substance-related disorder. If ≥ 3 of the 6 questions were answered positively (i.e. with “always,” “frequent,” “strong increase,” “slight increase,” or “yes,” depending on the question), the student was assigned to the “addictive behavior” group (AIU +); and if < 3 of the 6 questions were answered positively, to the “no addictive behavior” group (AIU −). We examined the presence ( +) or absence ( −) of addictive behavior in the following areas: general internet use (AIU + /AIU −), information searches (AIUi + /AIUi −), random browsing (AIUr + /AIUr −), gaming (AIUg + /AIUg −), social networking (AIUn + /AIUn −), online shopping (AIUs + /AIUs −), online pornography (AIUp + /AIUp −), and online gambling (AIUgb + /AIUgb −).

### Evaluation of current mental well-being

To assess current mental well-being, we incorporated the WHO-5 Well-being Index into the questionnaire. The index measures mental well-being in the past 2 weeks and is used as a screening tool for depression. The point values are summed, and raw values range from 0 (lowest well-being) to 25 (highest well-being). A raw value < 13 indicates a low level of well-being and that the person should undergo specific diagnostic tests for depression. The raw value is multiplied by four to give a percentage between 0 and 100 [93].

### Statistical analysis

The Lime Survey software saved the dataset directly in Excel and SPSS. Statistical analysis was performed with SPSS Statistics Versions 21 and 23 for Microsoft Windows. Descriptive data were presented as absolute and relative frequencies, mean, SD, and range. The two groups “addictive behavior” and “no addictive behavior” were compared with Chi-square and Mann–Whitney U tests; if the number of cases was too small, Fisher’s exact test was used. *P *values < 0.05 were considered to be significant.

## Results

### Study sample

A total of 522 (11.9%) students contacted by email filled out the questionnaires; however, 39 had to be excluded, because too many responses were missing (31 had started the questionnaire but not responded to any of the questions, and 8 had only completed the demographic section). Thus, questionnaires from 483 (11.0%) students were available for analysis (*n* = 31 [68.5%] women; *n* = 152 [31.5%] men). The mean (SD) age was 22.96 (4.68) years (range: 17–62), and the mean (SD) number of years of university education was 2.8 (1.75) years (range: 0–9 years). The majority of students (*n* = 435, 90.1%) were on track to graduate on time, but 48 (9.9%) were not.

### Comparison of sociodemographic characteristics between the AIU + and AIU − groups

The mean (SD) year of study was significantly lower in the AIU + group than in the AIU- group (2.48 [1.72] vs 2.91 [1.75], respectively; *P* = 0.013). However, none of the other sociodemographic characteristics differed significantly between the two groups.

### Internet use and sleep behavior

Almost the whole group (*n* = 479, 99.2%) used the internet (4 participants did not respond to the question). Of the internet users, 120 (25.1%) were classified as AIU + . Students in the whole group used the internet for a mean (SD) of 3.33 (2.42) h/d (range: 1–24 h/d). Just over half (*n* = 256, 53.4%) owned a smartphone, and *n* = 148 (30.6%) stated that they liked to eat their meals in front of the computer. Responses to the question, “How many times a day do you check your e-mail?” were as follows: less than 5 times, *n* = 292 (60.5%); 5–15 times, *n* = 145 (30.0%); 16–25 times, *n* = 27 (5.6%); and more than 25 times, *n* = 16 (3.3%).

The students’ sleep behavior differed between the semesters and semester breaks. During the semester, 53.4% got up between 6 and 8 AM; and 39.3%, between 8 and 10 AM. During the semester breaks, the values were 12.8% and 55.3%, respectively. During the semester, 66.7% went to bed between 10 PM and 12 AM; 25.1% between 12 and 2 AM; and 3.3% between 2 and 4 AM. During the semester breaks, the values were 36.0%, 51.1%, and 10%, respectively.

We found significant differences in the internet use behavior of these two groups in that the AIU + group spent more time each day on the internet (*P* < 0.001), were more likely to own a smartphone (*P* = 0.02) and to eat in front of the computer (*P* = 0.001), checked their email more often (*P* = 0.002), got up later during the semester breaks (*P* = 0.003) and went to bed later during the semester (*P* < 0.001) and semester breaks (*P* < 0.001). Regarding the time at which the students turned their computer on in the morning, 8.9% reported turning it on as soon as they got up; and 38.1%, within an hour of getting up; the time at which the students turned on their computers in the morning did not differ significantly between the two groups.

### Different areas of internet use

**Fig. 1:**
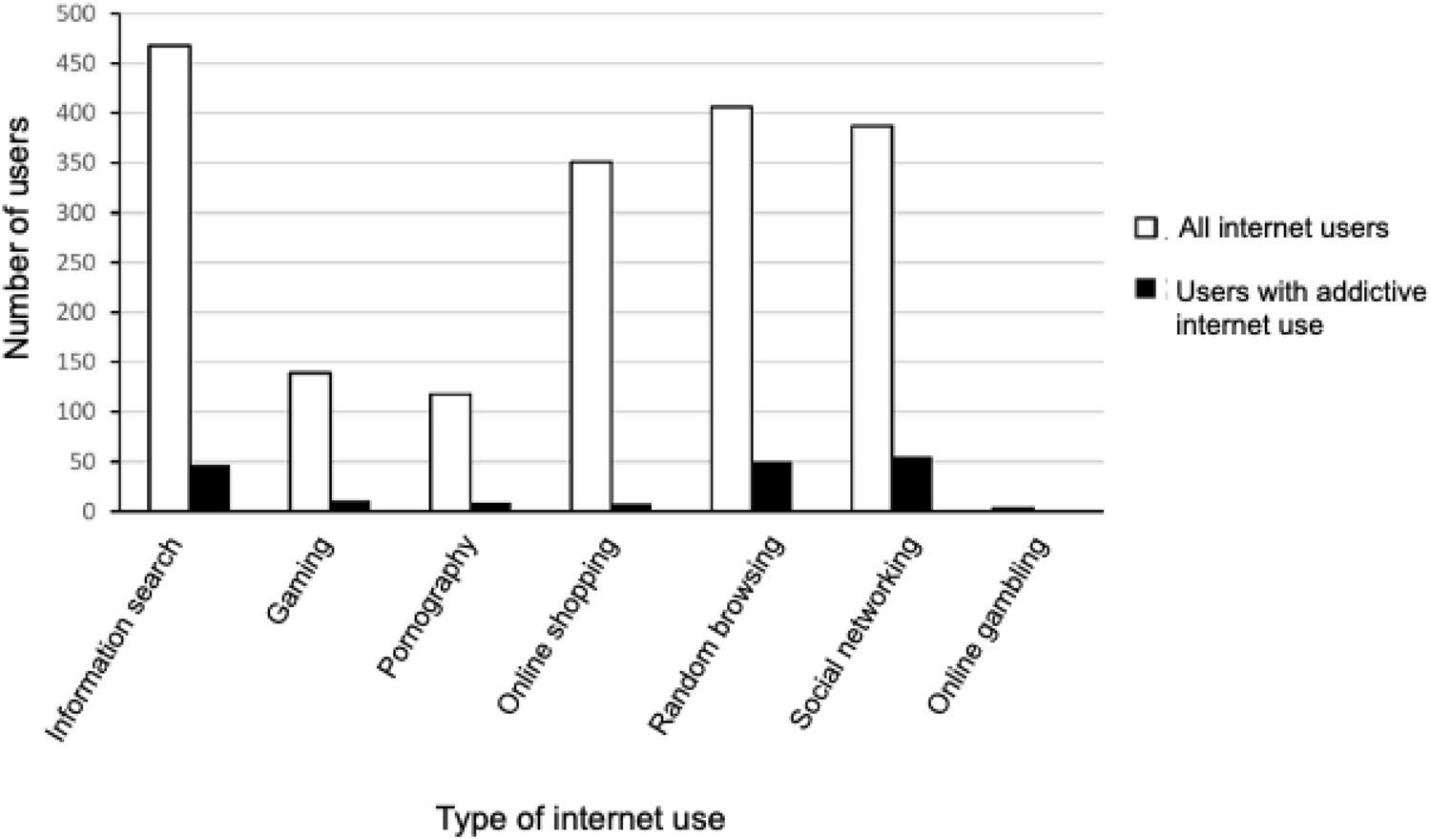
Proportion of university students (n = 479) who used the internet for the various reasons and the proportion who showed addictive internet use in each category of use

The most common reason for using the internet was to search for information (*n* = 468, 96.9%). Forty-seven (10.0%) of the internet users were classified as AIUi + in this area of use. Random browsing was the next most common reason for use (*n* = 406, 84.1%), and 50 (12.3%) of the internet users were classified as AIUr + . A total of *n* = 387 students (80.1%) used the internet for social networking, and 55 (14.2%) were classified as AIUn + . Online shopping was given as a reason for using the internet by *n* = 351 students (72.7%), and 8 (2.3%) were classified as AIUs + . Gaming was named by *n* = 139 students (28.8%), and 11 (7.9%) were classified as AIUg + ; and online pornography was named as a reason for internet use by *n* = 118 students (24.4%), and 9 (7.6%) were classified as AIUp + . The least common reason for using the internet was online gambling (*n* = 3 [0.6%]), and none of the users showed addictive behavior in this category. Figure 1 shows the proportion of students that used the internet for the various reasons and the proportion that showed AIU in each category, and Table 1 shows the mean hours per day that each group spent using the internet for the various reasons.

**Table 1:** Comparison of time spent using the internet for various reasons between the group of university students with addictive internet use and the group without addictive internet use (*N* = 473)

Reason for internet use	Internet use per day, mean (SD), h		*P* value
	Group with addictive internet use (*n* = 179)	Group without addictive internet use (*n* = 294)	
Information search	5.04 (4.77)	3.15 (1.93)	0.003
Random browsing	4.98 (4.54)	3.14 (1.96)	< 0.001
Social networking	4.48 (3.89)	3.19 (2.13)	0.003
Online shopping	3.25 (1.67)	3.34 (2.44)	0.774
Gaming	5.36 (3.47)	3.29 (2.38)	0.018
Online pornography	3.56 (1.88)	3.33 (2.44)	0.465
Online gambling	n/a	3.33 (2.42)	n/a

### Sociodemographic variables

The year of study was significantly lower in the AIUr + group than in the AIUr- group (*P* = 0.024) and in AIUn + than in AIUn − (*P* = 0.034), and the likelihood of being male was significantly higher in the AIUp + group than in the AIUp- group (*P* = 0.001). We found no other significant differences in year of study or sex in the remaining subgroups of areas of internet use and no significant differences in age or whether students were taking longer than normal for their studies between any of the subgroups.

### Well-being, social factors, and substance use

The associations between internet use, well-being, social factors, and substance use are described below for the AIU + and AIU − groups, as well as for the subgroups of internet use information search (AIUi + /AIUi −), random browsing (AIUr + /AIUr −), and social networking (AIUn + /AIUn −). The numbers of students with AIU in the areas of gaming (AIUg + , *n* = 11), online shopping (AIUs + , *n* = 8), online pornography (AIUp + : *n* = 9), and online gambling (AIUgb + : *n* = 0) were too small for statistical analysis.

### Well-being

**Fig. 2:**
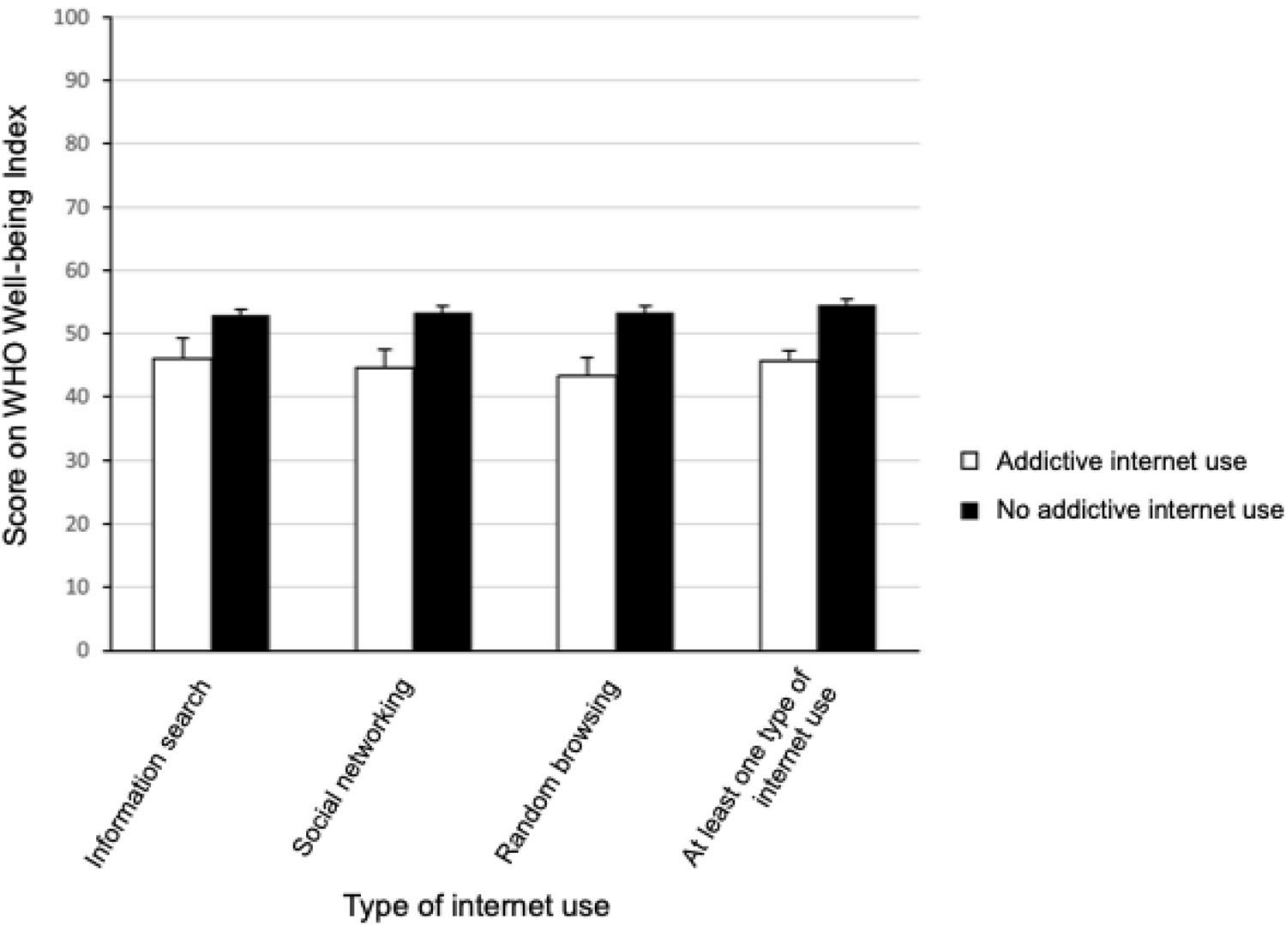
Comparison of the score on the WHO Well-being Index between university students with and without addictive internet use in various categories of internet use

The score on the WHO-5 Well-being Index was significantly lower in the AIU + group than in the AIU − group (mean [SEM], 45.59 [19.16] vs 54.51 [18.83], respectively; *P* < 0.001). This was also true for the AIUi + vs the AIUi − group (46.10 [21.46] vs 52.92 [18.96], respectively; *P* = 0.027), the AIUr + vs the AIUr − group (43.23 [20.32] vs 53.33 [18.90], respectively, *P* < 0.001), and the AIUn- vs AIUn + group (44.58 [21.10] vs 53.35 [18.80], respectively; *P* = 0.002) (Fig. 2).

### Social factors

We found no significant differences in social factors between the AIU + and AIU − groups, i.e. in the frequency of meeting friends, the number of hobbies not related to a computer, the time spent on these hobbies, the educational level of the mother and father, employment status, relationship status, whether they had met their partner on the internet, whether they had siblings, whether they had lived away from their parents, and the stress of their studies.

In the area of internet use for information search, students in the AIUi + group were significantly more often stressed by their studies than those in the AIUi- group (*P* < 0.01). We found no other significant differences in social factors between these two subgroups or between the random browsing subgroups (AIUr + /AIUr −) or social networking subgroups (AIUn + /AIUn −).

### Substance use

The AIU + and AIU − groups did not differ with respect to the frequency of smoking cigarettes, the number of cigarettes smoked daily, the frequency of alcohol use, amount of alcoholic drinks per week, frequency of being fully intoxicated in the previous month, illegal drug use, or the consumption of energy drinks (see Table 2).

**Table 2:** Comparison of substance use between university students with addictive internet use and those without addictive internet use in the categories of general internet use and internet use for random browsing and social networking

Type of substance use	General internet use			Internet use for random browsing			Internet use for social networking		
	AIU	No AIU	P value	AIU	No AIU	P value	AIU	No AIU	*P* value
Cigarette smoking, *n* = 454	n: 113 mean: 4,54 SD: 1,044	n: 341 mean: 4,57 SD: 0,994	0.822	*n*: 47 Mean: 4,34 SD: 1,290	*n*: 407 Mean: 4,58 SD: 0,966	0.277	*n*: 55 Mean: 4,49 SD: 1,086	*n*: 399 Mean: 4,57 SD: 0,995	0.542
Cigarettes smoked/d, mean (SD)	4.61 (4.65)	5.25 (5.94)	0.982	6.55 (4.85)	4.82 (5.67)	0.137	4.00 (5.31)	5.28 (5.63)	0.417
Alcohol, *n* = 454	n: 113 Mean: 3,35 SD: 0,894	n: 341 Mean: 3,40 SD: 0,880	0.804	*n*: 47 Mean: 3,34 SD: 0,788	*n*: 407 Mean: 3,39 SD: 0,894	0.803	*n*: 55 Mean: 3,18 SD: 0,819	*n*: 399 Mean: 3,41 SD: 0,889	0.074
Number of standard alcoholic drinks/wk, mean (SD)	6.66 (23.95)	8.05 (23.46)	0.259	8.54 (35.81)	7.59 (21.79)	0.208	5.57 (11.21)	8.02 (24.97)	0.614
Days fully intoxicated in recent months, mean (SD)	0.77 (3.82)	0.43 (0.95)	0,723	0.45 (0.775)	0.52 (2.18)	0.427	1.25 (5.39)	0.41 (0.93)	0.037*
Illegal drugs	13	5	0.417	7	0	0.183	6	5	0.653
Energy drinks	40	101	0.099	19	0	0.039*	20	28	0.264

^*^P < 0.05

We also found no differences in substance use between the AIUi + and AIUi − groups. In the random browsing groups, energy drinks were consumed significantly more often in the AIUr + group than in the AIUr − group (*P* = 0.039), and in the social networking groups, students in the AIUn + group had been fully intoxicated in the previous month significantly more often than students in the AIUn − group (*P* = 0.037; Table 2).

## Discussion

The present study surveyed internet use in 0.99% of LMU students in the winter semester 2012/2013. In one quarter of the sample, we identified AIU according to the study criteria, i.e. fulfilment of at least 3 of the ICD-10 diagnostic criteria for addiction disorders. Students classified as having AIU used the internet for significantly longer periods than those without AIU, but the only significant difference in demographic variables between the two groups was in the year of study, i.e. those with AIU were significantly less advanced in their studies. Regardless of whether or not participants were classified as having AIU, the most common reason for using the internet was information searches, followed by random browsing, social networking, and online shopping. Only about a quarter of the respondents used the internet for online gaming or pornography. The most common type of AIU was in social networking, followed by random browsing, information search, gaming, and online pornography, and addiction to online shopping or gambling was not common. These findings indicate that social networking, random browsing, information searches, gaming, and online pornography have the potential to be addictive. With the exception of online pornography, this finding was also reflected in significantly longer times spent on the internet, suggesting that patterns of use differ between different types of internet use. The only significant difference in sociodemographic variables was in online pornography, which was significantly more common among men. We did not identify any social factors as being possible risk factors for AIU in any of the different areas of internet use, and substance use also was not associated with AIU. The main finding was that subjective well-being was significantly lower in groups with AIU.

The high rate of internet use among respondents (99.2%) was expected and in line with earlier studies, e.g., a 2004 study that found a rate of 95.1% among youths (aged 12–18 years) in Norway [40]. The mean usage time in our study was 3.33 h per day, which is also similar to an earlier finding of 3.4 h per day [104]. Just over half (53%) of the respondents in our study had a smartphone, a rate largely consistent with results from other studies, e.g., 64% [92]; the 11% higher rate found by Smith in 2015 is not surprising given the fact that our study was conducted in 2012/2013. Even in 2011, only 35% of adult Americans owned a smartphone [92]. Nearly one-third of respondents indicated that they liked to eat their meals in front of the computer. Almost one-tenth said that they turned on the computer as soon as they got up, and more than a third had turned their computer on within an hour of getting up. These results reflect how important the internet is in students’ everyday lives. Internet use patterns indicated that a quarter of the respondents had AIU, which is higher than the rates of 12–15% found in other studies in students [14, 68, 110]. The fact that our study did not subdivide the participants with AIU may explain why our prevalence rates of AIU were relatively high. Prevalence rates vary greatly depending on various factors, such as sample, region and diagnostic instrument, and range from 0.2 [20] to 15.3% [68]. Alternative explanations for the high prevalence rate in our study may be that we used the ICD-10 classification, which overestimates addiction-like internet use, and that our study may have had a selection bias.

The prevalence rates for the individual areas of internet use are more congruent with those described in the literature. Compared with the AIU − group, the AIU + group spent significantly longer on the internet each day, a finding that is in agreement with other studies [57, 68]. Also, students in the AIU + group more often owned a smartphone and ate meals in front of the computer, and they checked their e-mail inboxes more frequently. The day–night rhythm differed between the two groups in that the AIU + group got up later than the AIU − group during the semester break and went to bed later during both the semester and the semester break. This finding illustrates the extent to which internet use changes daily routines and how essential the internet has become for some users. These results led us to hypothesize that the risk of AIU is increasing as internet use is becoming more closely integrated into our everyday lives. The finding that the AIU + group was more likely than the AIU − group to own a smartphone could mean that this factor also plays a role in AIU in general. Easier and more convenient internet access, such as on a cell phone, may further lower the threshold for internet use, which in turn may explain the higher incidence of AIU. This could be problematic for therapy for AIU, because it would make effective stimulus control, which could be an important therapeutic approach, difficult. However, this study was not able to prove causality. For example, having AIU may increase the likelihood of owning smartphone rather than being a consequence of having a smartphone.

The only demographic variable that differed significantly between the AIU + and AIU − students was the academic year, i.e. the AIU + students were significantly less advanced in their studies. This difference may be due to the fact that many students set their priorities differently at the beginning of their studies than towards the end, when graduation and subsequent employment are approaching. Other studies found a higher risk of AIU among adolescents than adults [58], but age was not associated with AIU in our study, perhaps because the majority of our participants were of a similar age. We also found no differences in the prevalence of AIU between men and women. This finding is in line with some studies, but contradicts others that found a significantly higher prevalence of AIU in men than in women [50, 68, 110].

Among the whole group of students, the internet was most commonly used for four of the seven specific areas, i.e. to search for information and for random browsing, social networking, and online shopping. A significantly smaller proportion of students used the internet for gaming or online pornography, and online gambling was rare. Information searches [74], social networking [75], and online shopping [86] are described in the literature as common areas of use of the internet. Random browsing has not yet been described in the literature, but it is mentioned by users in internet forums [83] and was a prevalent area of use in the current study. Although our study found that online gaming was less common than other categories, gaming is known to have high addictive potential [55]. The same is true for online pornography [100]. Despite the low internet use rates in these areas, they remain important in the context of AIU.

The largest proportion of AIU was in the area of social networking, followed by random browsing, information searches, gaming, and online pornography. Although many students used the internet for online shopping, only a small number were classified as having AIU in this category. The low rate of AIU for online shopping in our study may indicate that this type of internet use has a lower addictive potential, but our finding contrasts with other studies that showed a high addiction potential for online shopping among students [57]. The same study showed that social networking and gaming also have high addictive potential [57]. Noteworthy in this context is that AIU in the area of social networking is more common among women than men. In contrast, in the area of online pornography, AIU was more common in men than in women, which is in line with the findings of other studies [2, 10]. Online gambling and shopping were found to be less addictive than gaming and social networking [85].

With regard to the amount of time spent on the internet, we found significant differences between the AIU + and AIU − groups in the areas of random browsing, social networking, information searches, and gaming. Students with AIU in these areas spent significantly more time using the internet for these purposes than those who did not have AIU. This finding indicates that addictive behavior in these areas is particularly reflected in the amount of time spent on the internet. This is different for the areas of online shopping and online pornography, where we found no significant differences between AIU + and AIU − with regard to the duration of internet use. Therefore, we hypothesize that time spent on the internet is not necessarily a useful criterion per se for differentiating between AIU + and AIU − in general. However, other studies did find an association between addiction-like online shopping [39] and addiction-like use of online pornography [19] and the time spent using the internet for these purposes. The available results suggest that some areas of use are more time-intensive than others in AIU. Thus, duration of internet use may be useful for differentiating between people with and without AIU in specific areas of internet use, and future studies should examine the various areas of use separately.

We were surprised that the only sociodemographic variable that differed significantly between the two groups was the year of study with regard to random browsing, i.e., students in the AIUr + group were not as advanced in their studies as those in the AIUr − group. Reasons for this finding should be addressed in further studies.

Online shopping is not well studied to date, although studies identified female sex as a predisposing factor for compulsive and addictive shopping behavior [7, 17, 18]. We expected to find that sex was a relevant factor in the AIUs + group, but this was not the case.

Many studies have examined online gaming. The highest prevalence was found in adolescents [58] and among men [84]. We were unable to confirm these findings in our study, but this may have been due to our sample size or assessment tool. AIU in the area of gaming was shown to be associated with worse academic performance [15, 84], so one would expect that students in the AIUg + group were less likely to be on track to graduate on time. However, in the present study, we found no difference between the two groups regarding the likelihood of graduating on time.

The results of our study are in contrast to previous studies that found that AIU in the area of social networking was more prevalent among women [85]. The only significant difference between the AIUn + and AIUn − groups in our study was the year of study, i.e., students in the AIUn + group were less far advanced. An explanation for this finding may be that people with AIU in the area of social networking are more likely to discontinue their studies, so AIU is less likely to be found among more advanced students.

With regard to social factors, we found hardly any significant differences between those with AIU and those without. This finding contradicts studies which found that AIU has a negative effect on intrafamilial interactions, leisure activities, social contacts, and academic performance [28, 69,74, 80, 81, 84]. We found a significant difference only in the stress of studying in students with AIU in the area of information searches, i.e. these students were significantly more overwhelmed by their studies than those without AIU. This may be because excessive online searches for information are overwhelming for students who are struggling academically. However, causality is also unclear here. An alternative explanation could be that the overwhelming experience of studying leads to an excessive search for information online.

We found no significant differences in substance use between AIU + and AIU −. This contrasts with results from other studies, which suggested that AIU correlates with cannabis use [96]. The only significant difference was in the area of random browsing, where we found that the AIUr + group consumed energy drinks significantly more frequently than the AIUr − group. We would have expected to find such a result also in the area of gaming because energy drinks are widely advertised in the gaming scene. In the area of social networking, we found that the AIUn + group was fully intoxicated significantly more often than the AIUn- group, but we were unable to find a plausible explanation for this finding. Substance use disorders and AIU are similar on many levels [55], and people have argued that specific addictive behavior, such as substance use disorders, pathological gambling, and AIU, are merely different manifestations of the same underlying syndrome of addictive behavior [90]. Other studies showed a clear relationship between AIU and the use of illegal drugs [24] and harmful alcohol use [48]. The results of the present study contradict these findings in that we found no higher prevalence of substance use among students with AIU. Across all groups, students with AIU had significantly worse well-being than those without AIU. This result is consistent with the literature [20, 63] and shows that AIU goes hand in hand with reduced well-being and causes suffering. To date, the relationship between AIU and well-being is unclear. For example, AIU may reduce well-being or low well-being may facilitate AIU or both. Furthermore, both AIU and well-being may be influenced by other factors. Further research on this topic is warranted.

Our study has some limitations. First, we based the assessment of AIU on the ICD-10 criteria for substance use disorders. Thus, participants with three or more positive responses in one area of use were classified as having AIU in that area. We deliberately chose this approach, because results are barely comparable between studies as a consequence of the wide variety of assessment tools used [71, 104, 107]. At the time the questionnaire was prepared, we did not know of the plans to include internet gaming disorder as a condition for further study in DSM-5, so we were unable to use DSM-5 criteria to assess AIU. These new criteria appear to be useful for differentiating between people with and without addiction to online gaming, but criteria for areas of AIU other than online gaming are still lacking. Second, the length of our questionnaire (137 questions, requiring an estimated 15 min to complete) may have had a negative impact on the response rate, because studies have shown that above a certain questionnaire length the dropout rate increases and the quality of the responses decreases [25]. A processing time of 10 min is ideal [8]. Last, we can only extrapolate the findings in our sample to a limited extent to the whole population of students at LMU Munich, because we were only able to send the questionnaire to the approximately 10% of students who had agreed to take part in surveys. Thus, the study only included those students who were more motivated to actively participate in such studies, which may have had an impact on the results. We do not know whether the sociodemographic profile of the group of students who actively chose to take online surveys differed significantly from those who did not; therefore, we cannot exclude non-response bias and sample selectivity.

High prevalence rates of AIU in the group of students in this study and the associated suffering in the form of reduced well-being reflect the great importance of the phenomenon. The findings of this study may help to design specific support measures for students. Although the inclusion of internet gaming disorder in DSM-5 is a first step towards a better diagnostic classification of AIU, the DSM only highlights a small area of this growing problem. Similar to other research, the present study contains indications that areas of internet use other than gaming can also lead to addictive behavior with corresponding mental stress. Addictive behavior appears to differ between the different areas of use, so future research should take a more differentiated look at the various types of internet use to contribute to more accurate diagnoses and more specific treatment recommendations. Since the time of our study, the availability of smartphones has increased and students are required to complete more of their studies online, so the rate of AIU and associated symptoms may have increased. A follow-up study in a new cohort of students would therefore be of great interest.
